# Comparison of Two Symptom Checkers (Ada and Symptoma) in the Emergency Department: Randomized, Crossover, Head-to-Head, Double-Blinded Study

**DOI:** 10.2196/56514

**Published:** 2024-08-20

**Authors:** Johannes Knitza, Ragip Hasanaj, Jonathan Beyer, Franziska Ganzer, Anna Slagman, Myrto Bolanaki, Hendrik Napierala, Malte L Schmieding, Nizam Al-Zaher, Till Orlemann, Felix Muehlensiepen, Julia Greenfield, Nicolas Vuillerme, Sebastian Kuhn, Georg Schett, Stephan Achenbach, Katharina Dechant

**Affiliations:** 1 Institute for Digital Medicine University Hospital Giessen Philipps University Marburg Germany; 2 Department of Internal Medicine 3 Friedrich-Alexander University Erlangen-Nürnberg Universitätsklinikum Erlangen Erlangen Germany; 3 Deutsches Zentrum für Immuntherapie Friedrich-Alexander University Erlangen-Nürnberg Erlangen Germany; 4 Université Grenoble Alpes Grenoble France; 5 Department of Cardiology Friedrich-Alexander Universität Erlangen-Nürnberg Erlangen Germany; 6 Emergency and Acute Medicine and Health Services Research in Emergency Medicine Charité - Universitätsmedizin Berlin Berlin Germany; 7 Institute of General Practice and Family Medicine Charité - Universitätsmedizin Berlin Berlin Germany; 8 Institute of Medical Informatics Charité - Universitätsmedizin Berlin Berlin Germany; 9 Department of Medicine 1 Friedrich-Alexander University Hospital Erlangen University Erlangen-Nuremberg Erlangen Germany; 10 Centre for Health Services Research Brandenburg Brandenburg Medical School Rüdersdorf Germany; 11 Institut Universitaire de France Paris France; 12 Orange Labs & Université Grenoble Alpes Grenoble France

**Keywords:** symptom checker, triage, emergency, eHealth, diagnostic accuracy, apps, health service research, decision support system

## Abstract

**Background:**

Emergency departments (EDs) are frequently overcrowded and increasingly used by nonurgent patients. Symptom checkers (SCs) offer on-demand access to disease suggestions and recommended actions, potentially improving overall patient flow. Contrary to the increasing use of SCs, there is a lack of supporting evidence based on direct patient use.

**Objective:**

This study aimed to compare the diagnostic accuracy, safety, usability, and acceptance of 2 SCs, Ada and Symptoma.

**Methods:**

A randomized, crossover, head-to-head, double-blinded study including consecutive adult patients presenting to the ED at University Hospital Erlangen. Patients completed both SCs, Ada and Symptoma. The primary outcome was the diagnostic accuracy of SCs. In total, 6 blinded independent expert raters classified diagnostic concordance of SC suggestions with the final discharge diagnosis as (1) identical, (2) plausible, or (3) diagnostically different. SC suggestions per patient were additionally classified as safe or potentially life-threatening, and the concordance of Ada’s and physician-based triage category was assessed. Secondary outcomes were SC usability (5-point Likert-scale: 1=very easy to use to 5=very difficult to use) and SC acceptance net promoter score (NPS).

**Results:**

A total of 450 patients completed the study between April and November 2021. The most common chief complaint was chest pain (160/437, 37%). The identical diagnosis was ranked first (or within the top 5 diagnoses) by Ada and Symptoma in 14% (59/437; 27%, 117/437) and 4% (16/437; 13%, 55/437) of patients, respectively. An identical or plausible diagnosis was ranked first (or within the top 5 diagnoses) by Ada and Symptoma in 58% (253/437; 75%, 329/437) and 38% (164/437; 64%, 281/437) of patients, respectively. Ada and Symptoma did not suggest potentially life-threatening diagnoses in 13% (56/437) and 14% (61/437) of patients, respectively. Ada correctly triaged, undertriaged, and overtriaged 34% (149/437), 13% (58/437), and 53% (230/437) of patients, respectively. A total of 88% (385/437) and 78% (342/437) of participants rated Ada and Symptoma as very easy or easy to use, respectively. Ada’s NPS was –34 (55% [239/437] detractors; 21% [93/437] promoters) and Symptoma’s NPS was –47 (63% [275/437] detractors and 16% [70/437]) promoters.

**Conclusions:**

Ada demonstrated a higher diagnostic accuracy than Symptoma, and substantially more patients would recommend Ada and assessed Ada as easy to use. The high number of unrecognized potentially life-threatening diagnoses by both SCs and inappropriate triage advice by Ada was alarming. Overall, the trustworthiness of SC recommendations appears questionable. SC authorization should necessitate rigorous clinical evaluation studies to prevent misdiagnoses, fatal triage advice, and misuse of scarce medical resources.

**Trial Registration:**

German Register of Clinical Trials DRKS00024830; https://drks.de/search/en/trial/DRKS00024830

## Introduction

The number of emergency department (ED) visits is continuously increasing [1,2]. From 2009 to 2015, the number of ED outpatient care patients increased by 42% in Germany [1]. The significantly longer length of stay in ED due to the COVID-19 pandemic [2] aggravated the overcrowding of EDs [3]. Furthermore, the modern health care consumer has become accustomed to on-demand services. As a result, a significant number of nonurgent patients use emergency department services due to extended waiting times and limited operating hours of outpatient services [4]. ED crowding is associated with increased patient mortality, morbidity, longer stays, higher costs, medical errors, and staff burnout [4,5]. Multiple solutions to ED crowding have been introduced with varying degrees of success [4], one of which is optimizing ED input [6], by redirecting low-acuity patients to regular outpatient services.

Traditionally, acuity is determined on-site using triage systems with different performances [7]. Ideally, patients should undergo a quick and easily accessible online prescreening to assess their acuity and determine whether redirection to other health care services is appropriate. Demand for telemedicine services has increased due to the COVID-19 pandemic [8,9] and has also transformed emergency care [10]. While on-demand synchronous telephone and video consultations are cost-effective [11], new asynchronous automated services without personnel, such as symptom checkers (SCs), promise greater scalability and cost-effectiveness and are increasingly used by the public [9,12,13]. Implementation of such a preceding remote step-up (asynchronous, then synchronous) assessment successfully resolved 75% of acute care episodes at an American university hospital ED [10]. Winn et al [13], demonstrated that after consulting an SC, the urgency of patients’ intended level of care decreased in more than one-quarter of the cases among more than 150,000 patients; however, the study did not include any medical assessments.

In total, 2 of the most promising SCs according to recent studies [14-16] available in multiple languages, including German, are Ada and Symptoma. These underlying studies, however, were based on theoretical vignettes, small sample sizes, used nonrandomized trial designs, and were conducted by the respective manufacturers. Ada demonstrated the highest SC accuracy, only slightly inferior to general physicians in a recent vignette-based study, conducted by Ada [14]. In 2 vignette-based studies [15,16], conducted by Symptoma, Symptoma showed the highest SC accuracy compared with other SCs, including Ada. SC reviews repeatedly call for large-scale, prospective real-world studies [17,18]. Symptoma demonstrated an area under the curve of 0.74 to predict COVID-19 positivity in a large prospective study including 9133 people experiencing COVID-19–like symptoms [15]. This trial aimed to compare the diagnostic accuracy, safety, usability, and acceptance of 2 SCs (Ada and Symptoma) in patients presenting to a University Hospital emergency department.

## Methods

### Ethical Considerations

The study was approved by the Institutional Review Board of Erlangen University Hospital (approval number #106_19 B), and written informed consent was obtained from all study participants. This study was prospectively registered in the German Register of Clinical Trials (DRKS00024830). Data was pseudonymized and results were reported according to the CONSORT-EHEALTH (Consolidated Standards of Reporting Trials of Electronic and Mobile HEalth Applications and onLine TeleHealth) checklist [19]. Patients did not receive any compensation for their participation.

### Study Design and Participants

In this randomized, crossover, head-to-head, double-blinded study, patients presenting to the emergency department for internal medicine of the University Hospital Erlangen (Erlangen, Bavaria, Germany) were recruited. Eligible participants were aged 18 years or older. Participants were excluded if they were (1) isolated, (2) unstable, requiring immediate medical attention, or (3) unwilling or unable to give informed consent.

### Symptom Checkers

Ada and Symptoma were chosen because of widespread usage, broad coverage of symptoms, free accessibility, and promising, yet contradicting evidence [14-16]. Both SCs are class I medical devices. Both SCs ask users for general information, including age, sex, and current symptoms. Furthermore, questions are asked based on entered symptoms and answers. Both SCs then present disease suggestions and their likelihood. Ada additionally presents users with recommended actions to take (ie, call an ambulance). Ada was available as a native app and downloaded at the beginning of the study, and available updates were installed as soon as they were available. Symptoma was available as a web app.

### Procedures

Participants were randomized 1:1 to group 1 (completing Ada first, continuing with Symptoma) or group 2 (completing Symptoma first, continuing with Ada) by computer-generated block randomization. Assisting personnel were present to help with SC completion on sixth-generation iPad devices (Apple Inc.), if necessary, and to measure completion time. In a consecutive survey, participants rated SC usability and acceptance. ED staff and patients were blinded to SC suggestions. Final discharge diagnosis and patient demographics were recorded. Patients with 2 or more chronic conditions were defined as multimorbid [20]. Chief complaints were categorized using a frequently used list [21,22], including 35 different symptoms, originally published in a textbook [23].

### Outcomes

The primary outcome was the diagnostic accuracy of SCs. The secondary outcomes were SC usability and acceptance.

### Primary Outcome

Diagnostic accuracy was defined as concordance between the final discharge diagnosis and top-1 (D1) and up to top-5 (D5) SC diagnoses. SC diagnoses were restricted to a maximum of 5 suggestions. If patients were admitted as inpatients, this discharge diagnosis was used in place of the ED diagnosis.

Suggested SC diagnoses were blindly reviewed by 6 ED-experienced physicians (2 of whom were board-certified emergency physicians) who classified diagnostic concordance as (1) identical, (2) plausible, or (3) diagnostically different, following the methodology of Hautz et al [24] and Bastakoti et al [25]. In addition, they classified SC suggestions per patient as safe or potentially life-threatening. Multimedia Appendix 1 displays the applied classification, including examples. Participants were randomly assigned so that concordance was assessed by 1 physician from the University Hospital of Erlangen and 1 physician from the University Hospital of Berlin. To standardize assessment, 15 participants were assessed and discussed by all physicians before the actual evaluation. Interrater agreement, using Cohen κ, was moderate (Ada κ=0.54; Symptoma κ=0.45). In case of disagreement, discrepancies were resolved in discussions between physicians. Physicians were blinded to all data except SC diagnoses and final discharge diagnoses. Final discharge diagnoses were classified as (1) confirmed diagnosis, (2) suspected diagnosis, and (3) symptom or exclusion of a diagnosis, by the head of the ED.

### Secondary Outcomes

In addition, for Ada, the available SC action advice was compared with a triage categorization, assigned a posteriori, by consensus of 2 physicians, including the local head of the ED. We adopted the pragmatic and frequently followed [7,26] 4 triage–level categories, which are emergent, urgent, routine, and self-care (Multimedia Appendix 1).

SC usability was assessed using a 5-point Likert scale (1=very easy to use and 5=very difficult to use). Acceptance was assessed using the net promoter score (NPS) [27]. Using an 11-point numeric rating scale (0=not at all likely to 10=extremely likely), participants were asked how likely they would recommend the respective SC to a friend. Answers between 0 and 6 are summarized as detractors, 7 and 8 as passives, and 9 and 10 as promoters. The final NPS was calculated by subtracting the percentage of detractors from the percentage of promoters.

### Statistical Analysis

We evaluated the cumulative proportion of identical, plausible, or incorrect diagnostic suggestions by Ada and Symptoma with exact 95% CI. A subanalysis was carried out according to the level of urgency, randomization arm, and cases with a confirmed diagnosis. Odds ratios and 95% CI were calculated to compare the proportion of identical or plausible diagnoses. The relationship between comorbidity and correct diagnostic suggestions was investigated using point biserial correlation. SCs also report the estimated probability of the suggested top diagnosis. We calculated the mean of the estimated top diagnosis probability and 95% CI for identical, plausible, or diagnostically different diagnoses to investigate whether a higher average probability was estimated by the SCs for identical diagnoses. All analyses were conducted using SPSS (version 29; IBM Corp; Released 2022). The significance level was set at .05.

## Results

### Participants

**Figure 1:**
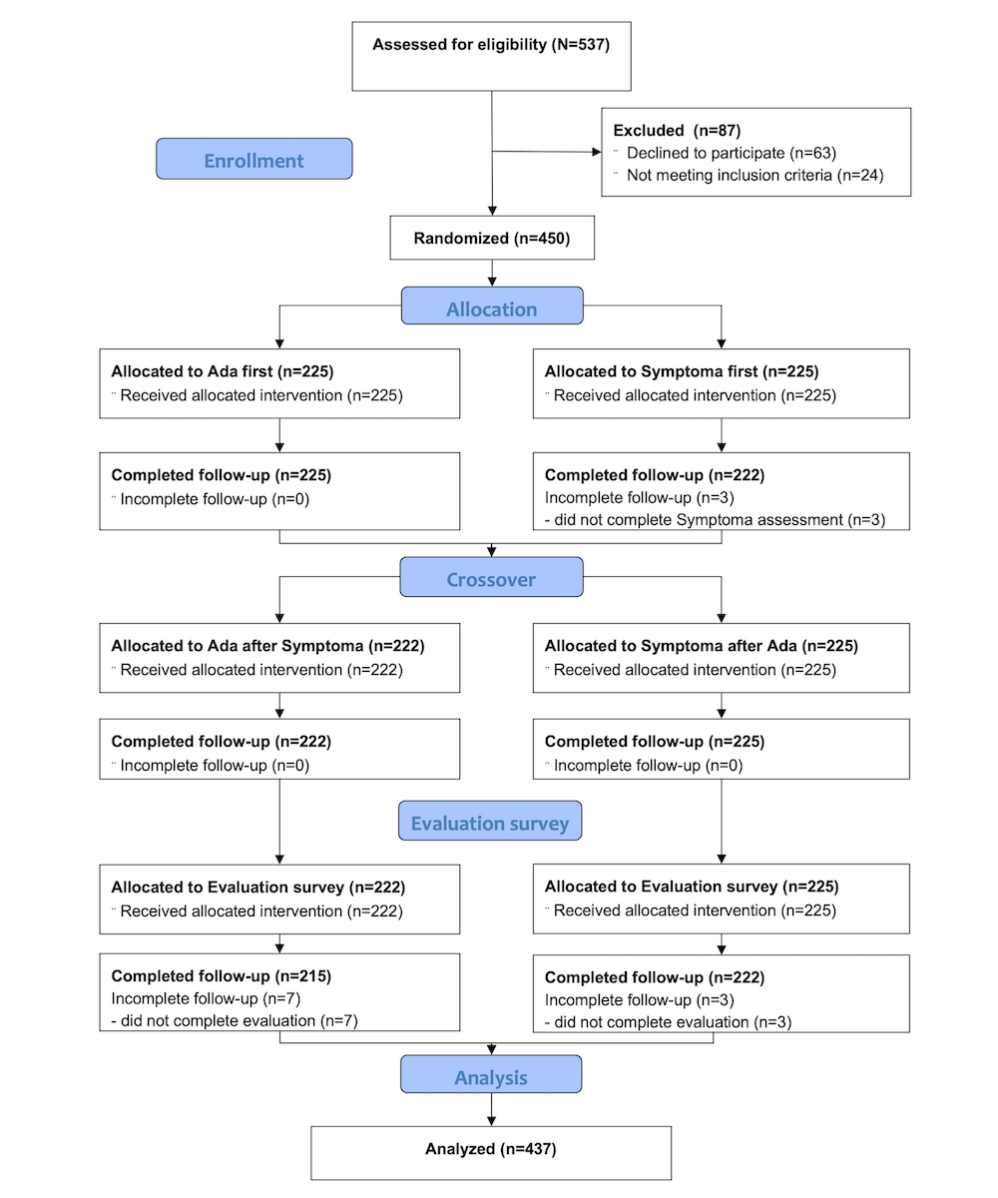
CONSORT (Consolidated Standards of Reporting Trials) diagram.

Between April 8, 2021, and November 15, 2021, a total of 537 participants were screened, of whom 450 were eligible and recruited and 437 were analyzed (Figure 1). Recruitment was limited to study nurse availability and daytime.

The mean age was 48.7 (SD 17.9) years. A total of 190/437 (43.5%) patients were female, 221/437 (50.6%) patients were multimorbid, and mean symptom severity was 4.0 (SD 2.7) out of 10 (Table 1). The most common chief complaint and final discharge diagnosis was chest pain (Table 1 and Multimedia Appendix 2). Final discharge diagnoses included 265/437 (61%) confirmed diagnoses, 32/437 (7%) suspected diagnoses, and 140/437 (32%) symptom or exclusion of diagnoses.

**Table 1:** Characteristics of study participants.

Characteristics				Total (N=437)
Age (years), mean (SD)				48.7 (17.9)
Female, n (%)				190 (43.5)
Multimorbid, n (%)				221 (50.6)
Symptom severity, mean (SD)				4.0 (2.7)
**Chief complaints, n (%)**				
	Chest pain			160 (36.6)
	Abdominal pain			76 (17.4)
	Dizziness			32 (7.3)
	Weakness			29 (6.6)
	Leg pain			10 (2.3)
	Other complaints			130 (29.7)
**Diagnoses, n (%)**				
	Chest pain			66 (15.1)
	Atrial fibrillation			33 (7.6)
	Collapse			27 (6.2)
	Hypertension			22 (5.0)
	Abdominal pain			21 (4.8)
	Gastritis			12 (2.7)
	Gastrointestinal bleeding			9 (2.1)
	Urinary tract infection			9 (2.1)
	NSTEMI^a^			8 (1.8)
	Other diagnoses			221 (50.6)
**Regular usage of mobile devices, n (%)**				
	Smartphone and tablet			163 (37.3)
	Smartphone only			219 (50.1)
	Tablet only			15 (3.4)
	None			40 (9.2)
**Previous symptom assessment, n (%)**				
		Online search engines	175 (40.0)	
		Symptom assessment website/app	47 (10.8)	
		Physician	149 (34.1)	

^a^NSTEMI: non–ST-segment myocardial infarction.

### Diagnostic Accuracy

**Figure 2:**
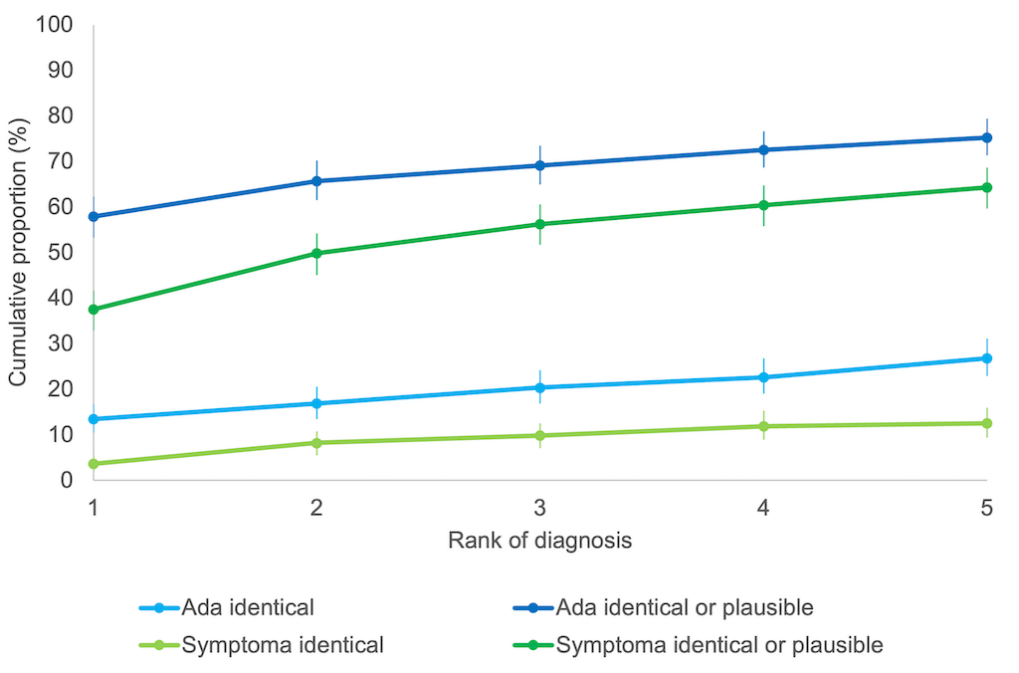
Cumulative proportion of identical and identical or plausible diagnoses by rank.

Overall, Ada made fewer suggestions compared with Symptoma (1777 vs 2167) and for several patients suggested only a top diagnosis (40 vs 1) or only one additional suggestion (81 vs 5). The overall odds ratio for an identical diagnosis using Ada compared with Symptoma was 2.54 (95% CI 1.78-3.62; *P*<.001) and for an identical or plausible diagnosis 1.69 (95% CI 1.26-2.27; *P*<.001). Figure 2 and Table 2 show the cumulative proportion of identical and plausible diagnoses with 95% CIs. Ada provided the identical diagnosis as the top diagnosis in 0.14 (95% CI 0.11-0.17) and within the top 5 diagnoses in 0.27 (95% CI 0.23-0.31) of patients, compared with Symptoma, which listed the identical top diagnosis in 0.04 (95% CI 0.02-0.05) and within the top 5 diagnoses in 0.13 (95% CI 0.09-0.16). An identical or plausible diagnosis was provided by Ada as the top diagnosis in 0.58 (95% CI 0.53-0.62) and within the top 5 diagnoses in 0.75 (95% CI 0.71-0.79), compared with Symptoma, which listed an identical or plausible diagnosis as the top diagnosis in 0.38 (95% CI 0.33-0.42) and within the top 5 diagnoses in 0.64 (95% CI 0.60-0.69). No significant differences were observed between randomization groups. Ada’s diagnostic performance was meaningfully better compared with Symptoma across all urgency levels and among cases with a confirmed diagnosis (Table 3). A point-biserial correlation was run to determine the relationship between top diagnostic probability and the number of patient comorbidities. No correlation was found to exist between the number of comorbidities and diagnostic accuracy for Ada (*r*_pb_=0.007, *n*=437; *P*=.88) or for Symptoma (*r*_pb_=–0.036, *n*=437; *P*=.46).

The mean reported top diagnosis probability did not correlate well with physicians’ classification (Table 4). Ada’s mean reported probability for an identical top diagnosis was 0.51 (95% CI 0.46-0.55) and for a diagnostically different diagnosis 0.40 (95% CI 0.38-0.43), compared with Symptoma, which showed a mean top diagnosis probability of 0.75 (95% CI 0.70-0.80) and 0.79 (95% CI 0.78-0.80) for diagnostically different diagnoses.

**Figure 3:**
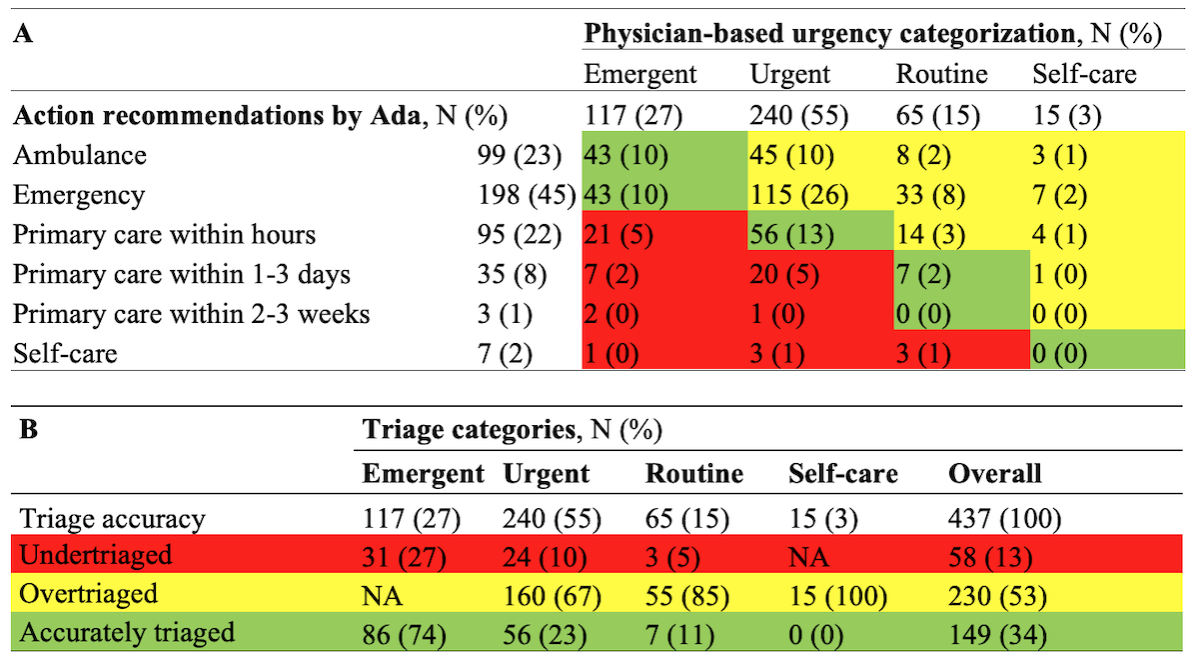
Confusion matrix of (A) Ada’s action recommendations and (B) overall triage accuracy compared with physician categorization.

Ada and Symptoma did not suggest potentially life-threatening diagnoses in 56/437 (13%) and 61/437 (14%), respectively. Compared with the physician-based classification, Ada overall triaged appropriately 149/437 (34%; Figure 3). A total of 74% (86/117) of emergent cases, 23% (56/240) of urgent cases, 11% (7/65) of routine cases, and 0% of self-care cases were adequately triaged by Ada.

**Table 2:** Cumulative proportion of identical or plausible diagnoses.

Rank	Ada (n=437)		Symptoma (n=437)	
	Identical, cumulative proportion (95% CI)	Identical or plausible, cumulative proportion (95% CI)	Identical, cumulative proportion (95% CI)	Identical or plausible, cumulative proportion (95% CI)
1	0.14 (0.11-0.17)	0.58 (0.53-0.62)	0.04 (0.02-0.05)	0.38 (0.33-0.42)
2	0.17 (0.14-0.21)	0.66 (0.62-0.70)	0.08 (0.05-0.11)	0.50 (0.45-0.54)
3	0.20 (0.17-0.24)	0.69 (0.65-0.73)	0.10 (0.07-0.13)	0.56 (0.52-0.61)
4	0.23 (0.19-0.27)	0.72 (0.69-0.77)	0.12 (0.9-0.15)	0.60 (0.56-0.65)
5	0.27 (0.23-0.31)	0.75 (0.71-0.79)	0.13 (0.09-0.16)	0.64 (0.60-0.69)

**Table 3:** The proportion of identical or plausible diagnoses of Ada and Symptoma according to case classification.

	Ada (n=437)							Symptoma (n=437)				
	Top diagnosis			Top 5 diagnoses			Top diagnosis				Top 5 diagnoses	
Case classification	Identical, proportion (95% CI)	Identical or plausible, proportion (95% CI)	Identical, proportion (95% CI)		Identical or plausible, proportion (95% CI)	Identical, proportion (95% CI)			Identical or plausible, proportion (95% CI)	Identical, proportion (95% CI)		Identical or plausible, proportion (95% CI)
Emergent (n=117)	0.10 (0.05-0.16)	0.61 (0.51-0.69)	0.20 (0.13-0.27)		0.74 (0.67-0.82)	0.03 (0.01-0.07)			0.40 (0.31-0.49)	0.10 (0.05-0.15)		0.74 (0.66-0.81)
Urgent (n=240)	0.15 (0.11-0.20)	0.55 (0.49-0.62)	0.32 (0.27-0.37)		0.76 (0.70-0.82)	0.04 (0.02-0.06)			0.36 (0.30-0.43)	0.15 (0.11-0.20)		0.60 (0.54-0.67)
Routine (n=65)	0.12 (0.05-0.20)	0.60 (0.48-0.72)	0.22 (0.11-0.31)		0.71 (0.60-0.82)	0.05 (0.00-0.11)			0.35 (0.23-0.48)	0.09 (0.03-0.17)		0.62 (0.49-0.72)
Self-care (n=15)	0.13 (0.00-0.33)	0.67 (0.40-0.87)	0.27 (0.07-0.53)		0.87 (0.67-1.00)	0.00 (0.00-0.00)			0.47 (0.20-0.73)	0.07 (0.00-0.20)		0.67 (0.47-0.87)
Confirmed diagnosis (n=265)	0.17 (0.13-0.21)	0.52 (0.46-0.58)	0.35 (0.33-0.38)		0.8 (0.6-0.9)	0.05 (0.03-0.08)			0.3 (0.25-0.36)	0.32 (0.29-0.34)		0.4 (0.3-0.5)

**Table 4:** Mean reported top diagnosis probability and 95% CI for identical, plausible, or diagnostically different diagnoses.

Mean diagnostic probability	Ada (n=437)	Symptoma (n=437)
Identical, mean (95% CI)	0.51 (0.46-0.55)	0.75 (0.70-0.80)
Plausible, mean (95% CI)	0.42 (0.40-0.44)	0.81 (0.80-0.81)
Diagnostically different, mean (95% CI)	0.40 (0.38-0.43)	0.79 (0.78-0.80)

### Usability and Acceptance

A total of 385/437 (88%) and 342/437 (78%) patients rated Ada and Symptoma as very easy or easy to use, respectively.

Median completion time for Ada was 7 (IQR 5-9) and 5 (IQR 3-6) minutes for Symptoma. A total of 114/437 (26%) and 113/437 (26%) patients requested help from study personnel to use Ada and Symptoma, respectively. Ada’s NPS was –34 due to 239/437 (55%) detractors and 93/437 (21%) promoters. Symptoma’s NPS was –47 with 275/437 (63%) detractors and 70/437 (16%) promoters.

## Discussion

To the best of our knowledge, this is the first and largest prospective head-to-head trial comparing the diagnostic accuracy, usability, and acceptance of 2 SCs (Ada and Symptoma), used by patients themselves. Our results elucidate that the diagnostic accuracy of both SCs was substantially lower compared with the final discharge diagnosis by clinicians.

Ada made fewer suggestions but reported an identical or plausible diagnosis significantly more often compared with Symptoma. This observed trend was independent of patient urgency level. One reason for this difference may be that Ada had more time to gather information, as the average completion time was 2 minutes longer. In line with a previous observation [28], the mean reported probability of the top diagnosis was misleading. Symptoma’s mean reported probability for diagnostically different diagnoses was actually higher than for identical diagnoses (79% vs 75%). Potentially life-threatening diagnoses were missed by both SCs equally often. Patients rated Ada as easier to use and were more likely to recommend it to other patients compared with Symptoma.

The landmark study by Semigram et al [18], benchmarking 23 symptom checkers using 45 vignettes, reported diagnostic accuracy of 34% for the top diagnosis and 51% for the top 3 diagnoses. Due to the theoretical and vignette-based nature of the study, direct comparison of our results with those of Semigran et al [18], is limited. The observed diagnostic inferiority of SCs compared with physicians is in line with previous studies [14,29,30]. Gilbert et al [14] reported an accuracy of 71% for Ada compared with general physicians with 82% based on case vignettes. In a previous rheumatology-based randomized controlled trial investigating Ada’s accuracy used by patients themselves, we observed a diagnostic accuracy of 43% (D1) and 54% (D5) regarding the detection of inflammatory rheumatic diseases [30]. Faqar-Uz-Zaman et al [29] reported results from a similar study investigating Ada in the ED in patients with abdominal pain. Ada suggested the discharge diagnosis in 52% (D5), compared with 81% by ED physicians. The authors suggested that physicians using the SC suggestions could theoretically improve physician’s accuracy by 10%. Martinez-Franco et al [31] reported a significantly higher accuracy in general physicians using an SC (DXplain) compared with a group without the tool.

Comparing the accuracy of SCs and physicians should be done carefully, as physicians had access to substantially more information including data from laboratory tests and imaging.

Previous studies have also reported diagnostic discrepancies between the initial ED diagnosis and the final discharge diagnosis [24,25]. It has also been shown that the diagnostic accuracy of physicians was lower than that of Ada when limited to symptom-related medical history [32]. The focus of this study was therefore to compare 2 SCs with each other, used by the same patient. To exclude a potential bias of the order of SC usage, patients were randomized.

Schmieding et al [33] showed that no symptom checker among 22 outperformed laypersons in deciding whether emergency care or self-care was adequate and that triage accuracy did not improve after 5 years, missing >40% of emergencies. Our study confirms results from previous studies [18,34] reporting that emergency cases are triaged more accurately than less-urgent cases. The percentage of undertriaged patients (13%) was slightly higher compared with a previous trial (9%) that investigated Ada in an interdisciplinary University Hospital ED [35].

In the study by Miller et al [36], 98% of patients reported Ada as very easy or quite easy to use, compared with 88% in this study. In an expert heuristic review of chatbots, Ada and Symptoma received an overall rating of 6.3/12 and 7.0/12, respectively [37]. The negative NPS indicates poor acceptance and contrasts the previously reported rate of 73% (440/600) [12] and 85% (444/520) [36] of patients who would recommend Ada. The 2 previous studies did not use the NPS but used a binary (Yes/No) approach and were based in a primary care and rheumatology setting. The negative NPS also contrasts with the positive Ada ratings on the German Apple App Store (4.7/5) and Google Play Store (4.6/5). We believe that the rather strict NPS rating system (only 9 and 10 counted as promoters) is the main reason for the difference.

This study has several strengths and limitations. Suggested SC diagnoses were blindly reviewed by physicians and patients were blinded to SC reports. For each case, 1 of the 2 assessors came from a completely different center (Charité, Berlin) to ensure a maximum of objectivity. The multidisciplinary study team involving nursing staff, as well as dedicated health service researchers, represents a strength of the study. The cardiology focus and single-center nature of the study limit generalizability. Moderate interrater agreement represents a common study limitation. We did not prespecify an effect size nor carried out a sample size calculation; however, our sample size is similar to the 2 largest SC studies [38,39] in an ED setting. A limitation to generalizability is the rapid pace of digital diagnostic advancements. This is due to the continuous updates of SCs and the emergence of powerful large language models, such as ChatGPT (OpenAI), which provide new diagnostic decision support [40,41]. A potential bias was that analysis was restricted to the top 5 diagnoses and Symptoma offers up to 30 suggestions. Furthermore, Symptoma’s guidelines specifically advise against its use in emergencies. The large size of the study, head-to-head nature, manufacturer independence, real-world setting, and high percentage of emergent patients represent strengths of the study.

### Conclusions

The accuracy and safety of symptom checkers appear inferior to a complete physician-based assessment. A substantial number of potentially life-threatening diagnoses were missed by both symptom checkers and the high number of patients undertriaged by Ada is alarming. Ada demonstrated a significantly higher diagnostic accuracy, was easier to use, and overall, better rated compared with Symptoma.

## Appendix

Multimedia Appendix 1 Diagnostic accuracy, safety, and urgency categorization with examples.

Multimedia Appendix 2 Final discharge diagnoses.

Multimedia Appendix 3 CONSORT-eHEALTH checklist (V 1.6.1).

## Abbreviations

CONSORT-EHEALTH: Consolidated Standards of Reporting Trials of Electronic and Mobile HEalth Applications and onLine TeleHealth
ED: emergency department
NPS: net promoter score
SC: symptom checker
