# Supplementary material for: Comparison of Two Symptom Checkers (Ada and Symptoma) in the Emergency Department: Randomized, Crossover, Head-to-Head, Double-Blinded Study
Source: J Med Internet Res. 2024 Aug 20;26:e56514. doi: 10.2196/56514 (PMC11372320; doi:10.2196/56514)
Supplement: Multimedia Appendix 1 [file jmir_v26i1e56514_app1.docx]

**Table S1. (A) Diagnostic accuracy and (B) safety categorization including symptom checker and final discharge diagnosis examples adapted from [24,25].**

| **A** |  | Example | |
| --- | --- | --- | --- |
| **Concordance rating** | **Description** | **Symptom checker diagnosis** | **Final discharge diagnosis** |
| Identical | Diagnoses are absolutely identical | Atrial fibrillation | Atrial fibrillation |
| Plausible | Very similar diagnosis that shares many symptoms with actual diagnosis and is plausible | Angina pectoris | Non-ST-segment myocardial infarction |
| Plausible | Symptom checker diagnosis is plausible and more precise | Atrial fibrillation | Chest pain |
| Plausible | Symptom checker diagnosis is plausible however less precise | Chest pain | Atrial fibrillation |
| Diagnostically different | Symptom checker diagnosis is implausible | Urinary tract infection | Non-ST-segment myocardial infarction |
| **B** |  |  |  |
|  |  | Example | |
| **Safety** | **Description** | **Symptom checker diagnosis** | **Final discharge diagnosis** |
| Danger | Final discharge diagnosis is potentially life-threatening and symptom checker does not include any potentially life-threatening diagnoses | Panic attack | Non-ST-segment myocardial infarction |

**Table S2. Categorization criteria used to determine acuity of patients adapted from [7,26].**

| **Categorization of acuity** | **Information from patient medical records** |
| --- | --- |
| Emergency | Patient required immediate treatment or was unstable, work-up or monitoring usually only available in hospital |
| Urgency | Patient's diagnosis was not life or limb threatening, but required timely assessment by an emergency physician or primary care provider within the same day |
| Routine | Management was wholly within scope of an outpatient family physician practice. Minimal risk for significant harm would result from delay in providing the treatment administered by the physician |
| Home | Management advice was given to the patient for an issue that did not require a prescription, referral, or further diagnostic testing |
