# Supplementary material for: Comparison of Two Symptom Checkers (Ada and Symptoma) in the Emergency Department: Randomized, Crossover, Head-to-Head, Double-Blinded Study
Source: J Med Internet Res. 2024 Aug 20;26:e56514. doi: 10.2196/56514 (PMC11372320; doi:10.2196/56514)
Supplement: Multimedia Appendix 2 [file jmir_v26i1e56514_app2.docx]

**Table S1. Final discharge diagnoses according to the International Classification of Disease 10th Revision (ICD-10).**

| ICD-10 | | | N (%) | |
| --- | --- | --- | --- | --- |
| R | 7 | 4 | 66 | (15.1) |
| I | 48 | 0 | 33 | (7.6) |
| R | 55 |  | 27 | (6.2) |
| I | 10 | 91 | 22 | (5.0) |
| R | 10 | 4 | 21 | (4.8) |
| K | 29 | 7 | 12 | (2.7) |
| K | 92 | 2 | 9 | (2.1) |
| N | 39 | 0 | 9 | (2.1) |
| T | 75 | 4 | 9 | (2.1) |
| I | 21 | 4 | 8 | (1.8) |
| I | 48 | 3 | 8 | (1.8) |
| I | 50 | 9 | 7 | (1.6) |
| R | 10 | 3 | 8 | (1.8) |
| B | 99 |  | 6 | (1.4) |
| I | 20 | 0 | 6 | (1.4) |
| I | 47 | 1 | 6 | (1.4) |
| I | 48 | 4 | 6 | (1.4) |
| N | 20 | 9 | 6 | (1.4) |
| T | 78 | 2 | 6 | (1.4) |
| K | 51 | 9 | 5 | (1.1) |
| I | 21 | 3 | 4 | (0.9) |
| I | 25 | 9 | 4 | (0.9) |
| I | 26 | 9 | 4 | (0.9) |
| I | 40 | 9 | 4 | (0.9) |
| I | 49 | 3 | 4 | (0.9) |
| K | 85 | 9 | 4 | (0.9) |
| N | 17 | 9 | 4 | (0.9) |
| R | 0 | 1 | 3 | (0.7) |
| R | 42 |  | 4 | (0.9) |
| I | 20 | 9 | 3 | (0.7) |
| I | 21 | 9 | 3 | (0.7) |
| I | 44 | 2 | 3 | (0.7) |
| K | 52 | 9 | 3 | (0.7) |
| R | 6 | 0 | 3 | (0.7) |
| R | 53 |  | 3 | (0.7) |
| A | 9 | 9 | 2 | (0.5) |
| C | 25 | 9 | 2 | (0.5) |
| E | 11 | 91 | 2 | (0.5) |
| E | 14 | 61 | 2 | (0.5) |
| E | 87 | 6 | 2 | (0.5) |
| F | 41 | 0 | 2 | (0.5) |
| I | 80 | 2 | 2 | (0.5) |
| J | 15 | 9 | 2 | (0.5) |
| K | 35 | 8 | 2 | (0.5) |
| K | 50 | 9 | 2 | (0.5) |
| K | 56 | 7 | 2 | (0.5) |
| K | 57 | 1 | 2 | (0.5) |
| K | 59 | 0 | 2 | (0.5) |
| K | 80 | 2 | 2 | (0.5) |
| L | 52 |  | 2 | (0.5) |
| M | 2 | 19 | 2 | (0.5) |
| M | 6 | 0 | 2 | (0.5) |
| M | 35 | 3 | 2 | (0.5) |
| M | 79 | 16 | 2 | (0.5) |
| M | 79 | 63 | 2 | (0.5) |
| R | 11 |  | 2 | (0.5) |
| R | 17 | 9 | 2 | (0.5) |
| A | 41 | 9 | 1 | (0.2) |
| C | 19 |  | 1 | (0.2) |
| C | 34 | 9 | 1 | (0.2) |
| C | 64 |  | 1 | (0.2) |
| C | 78 | 2 | 1 | (0.2) |
| C | 80 | 9 | 1 | (0.2) |
| C | 82 | 9 | 1 | (0.2) |
| D | 50 | 9 | 1 | (0.2) |
| D | 57 | 0 | 1 | (0.2) |
| D | 68 | 9 | 1 | (0.2) |
| D | 69 | 4 | 1 | (0.2) |
| D | 73 | 3 | 1 | (0.2) |
| E | 3 | 9 | 1 | (0.2) |
| E | 5 | 9 | 1 | (0.2) |
| F | 45 | 3 | 1 | (0.2) |
| G | 57 | 9 | 1 | (0.2) |
| G | 73 | 7 | 1 | (0.2) |
| I | 20 | 8 | 1 | (0.2) |
| I | 42 | 0 | 1 | (0.2) |
| I | 45 | 6 | 1 | (0.2) |
| I | 47 | 9 | 1 | (0.2) |
| I | 49 | 4 | 1 | (0.2) |
| I | 80 | 9 | 1 | (0.2) |
| I | 80 | 89 | 1 | (0.2) |
| I | 87 | 21 | 1 | (0.2) |
| I | 95 | 9 | 1 | (0.2) |
| J | 44 | 1 | 1 | (0.2) |
| J | 45 | 9 | 1 | (0.2) |
| J | 84 | 9 | 1 | (0.2) |
| J | 86 | 9 | 1 | (0.2) |
| J | 90 |  | 1 | (0.2) |
| J | 93 | 9 | 1 | (0.2) |
| K | 21 | 9 | 1 | (0.2) |
| K | 31 | 5 | 1 | (0.2) |
| K | 57 | 4 | 1 | (0.2) |
| K | 57 | 9 | 1 | (0.2) |
| K | 71 | 0 | 1 | (0.2) |
| K | 74 | 6 | 1 | (0.2) |
| K | 75 | 4 | 1 | (0.2) |
| K | 80 | 1 | 1 | (0.2) |
| K | 80 | 3 | 1 | (0.2) |
| K | 80 | 80 | 1 | (0.2) |
| K | 81 | 0 | 1 | (0.2) |
| K | 83 | 0 | 1 | (0.2) |
| K | 85 | 81 | 1 | (0.2) |
| L | 98 | 9 | 1 | (0.2) |
| M | 10 | 9 | 1 | (0.2) |
| M | 45 | 9 | 1 | (0.2) |
| M | 47 | 9 | 1 | (0.2) |
| M | 79 | 17 | 1 | (0.2) |
| M | 79 | 24 | 1 | (0.2) |
| Q | 61 | 9 | 1 | (0.2) |
| R | 14 |  | 1 | (0.2) |
| R | 18 |  | 1 | (0.2) |
| R | 51 |  | 1 | (0.2) |
| S | 2 | 2 | 1 | (0.2) |
| S | 6 | 0 | 1 | (0.2) |
| T | 18 | 9 | 1 | (0.2) |
| T | 78 | 4 | 1 | (0.2) |
| T | 86 | 10 | 1 | (0.2) |
